# Transcriptome Analysis of Cotton (*Gossypium hirsutum* L.) Genotypes That Are Susceptible, Resistant, and Hypersensitive to Reniform Nematode (*Rotylenchulus reniformis*)

**DOI:** 10.1371/journal.pone.0143261

**Published:** 2015-11-16

**Authors:** Ruijuan Li, Aaron M. Rashotte, Narendra K. Singh, Kathy S. Lawrence, David B. Weaver, Robert D. Locy

**Affiliations:** 1 Department of Biological Sciences, Auburn University, Auburn, Alabama, United States of America; 2 Department of Entomology and Plant Pathology, Auburn University, Auburn, Alabama, United States of America; 3 Department of Crop, Soil and Environmental Sciences, Auburn University, Auburn, Alabama, United States of America; James Hutton Institute, UNITED KINGDOM

## Abstract

Reniform nematode is a semi-endoparasitic nematode species causing significant yield loss in numerous crops, including cotton (*Gossypium hirsutum* L.). An RNA-sequencing analysis was conducted to measure transcript abundance in reniform nematode susceptible (DP90 & SG747), resistant (BARBREN-713), and hypersensitive (LONREN-1) genotypes of cotton (*Gossypium hirsutum* L.) with and without reniform nematode infestation. Over 90 million trimmed high quality reads were assembled into 84,711 and 80, 353 transcripts using the *G*. *arboreum* and the *G*. *raimondii* genomes as references. Many transcripts were significantly differentially expressed between the three different genotypes both prior to and during nematode pathogenesis, including transcripts corresponding to the gene ontology categories of cell wall, hormone metabolism and signaling, redox reactions, secondary metabolism, transcriptional regulation, stress responses, and signaling. Further analysis revealed that a number of these differentially expressed transcripts mapped to the *G*. *raimondii* and/or the *G*. *arboreum* genomes within 1 megabase of quantitative trait loci that had previously been linked to reniform nematode resistance. Several resistance genes encoding proteins known to be strongly linked to pathogen perception and resistance, including LRR-like and NBS-LRR domain-containing proteins, were among the differentially expressed transcripts mapping near these quantitative trait loci. Further investigation is required to confirm a role for these transcripts in reniform nematode susceptibility, hypersensitivity, and/or resistance. This study presents the first systemic investigation of reniform nematode resistance-associated genes using different genotypes of cotton. The candidate reniform nematode resistance-associated genes identified in this study can serve as the basis for further functional analysis and aid in further development of reniform a nematode resistant cotton germplasm.

## Introduction

Reniform nematodes (RN, *Rotylenchlus reniformis*) are semi-endoparasitic nematode species causing significant yield loss in cotton [1]. At present genetic sources of resistance to RN in cotton are limited, and there are no RN tolerant/resistant commercial varieties available.

Successful RN parasitism is contingent on establishment of a syncytium, which serves as the sole nutrient source on which RN live. Nematode secretions injected through their stylet, a specialized needle-like mouthpart, are essential in syncytium initiation and maintenance [2, 3]. To date, several peptide sequences homologous to other sedentary plant parasitic nematode effectors have been identified from RN expressed sequence tag (EST) assemblies [4]. However, none of these have been experimentally studied.

A number of studies have been conducted using microarray or RNA-sequencing technology to characterize plant responses to sedentary plant endo-parasitic nematodes including root knot nematodes (RKN, *Meloidogyne* spp.) and cyst nematodes (CN, *Globodera* and *Heterodera* spp.) [5–13]. Based on the results from gene expression, molecular, and physiological studies, it was proposed that the host plant responses to nematodes rely on the coordination of different resistance mechanisms including specific resistance genes or proteins, several plant hormone pathways, and reactive oxygen species (ROS) that are generated in response to nematode attack [14]. These resistance-related elements can be viewed as an integrated signaling network involving “crosstalk” between elements leading to regulation mediated by transcription factors and small RNAs (sRNAs) at the transcriptional, posttranscriptional, and/or translational levels [14].

Several resistance genes (*R*-genes) that confer resistance to a variety of plant parasitic nematodes have been identified and cloned (see reference [14] for review). Most of these cloned *R*-genes were predicted to encode canonical intracellular R-protein receptors that contain a nucleotide-binding site (NBS) and a leucine-rich repeat (LRR) domain [14]. Intracellular NBS-LRR-type R-protein receptors and extracellular LRR domain-containing proteins are known to recognize invading pathogen elements and trigger plant innate immunity responses [15].

Plant innate immunity responses mediated through R-protein receptors often trigger the induction of hypersensitive responses (HR) [15]. HR involves a localized programmed cell death (PCD) response and/or generation of ROS, typically H_2_O_2_, at the pathogen infection site [15]. While the main purpose of PCD is to prevent the spread of the pathogen, rapid generation of ROS at the pathogen infection sites can not only trigger PCD locally, but can also be transferred systemically in a cell-to-cell auto-propagating manner and participate in systemic acquired resistance [16, 17].

Upland cotton, *G*. *hirsutum*, is a natural allotetraploid species that likely arose from interspecific hybridization between ancestral diploid species having an A-like genome (present day *G*. *arboreum*) and a D-like genome (present day *G*. *raimondii*) [18]. In 2012, two groups separately published assembled *G*. *raimondii* whole genome sequences [19, 20], and the draft genome of *G*. *arboreum* became available in April 2014 [21]. In the absence of a *G*. *hirsutum* complete genome sequence, the genomes of *G*. *arboreum* and *G*. *raimondii* afford the best available resources for genome-wide transcriptome analysis of *G*. *hirsutum*.

Two cotton-breeding lines with resistance to RN, LONREN-1 and LONREN-2 were originally released by the USDA [22]. In these lines, RN resistance was transferred to *G*. *hirsutum* from the wild diploid species, *G*. *longicalyx*, which is apparently immune to RN [22]. A codominant simple sequence repeat (SSR) BNL3279_114 marker was used to follow introgression of the RN resistance quantitative trait locus (QTL) *Ren* ^*lon*^ into *G*. *hirsutum* [22]. However, root necrosis and a progressive decrease in root mass, typical of an HR, were observed on the two LONREN lines with increased RN inoculum levels [23].

BARBREN-713 was later released by the USDA as another RN resistance genotype, based on its performance in RN resistance trials and promising agronomic potential [24]. BARBREN-713 was developed by crossing and backcrossing *G*. *barbadense* (tetraploid) accession GB713, an RN resistant line, with the RKN resistant cultivar Acala Nem-X [24]. The RN resistance of BARBREN-713 is primarily due to a homozygous QTL locus *Ren* ^*barb2*^ flanked by SSR markers BNL3279_105 and BNL4011_155. However, QTL *Ren* ^*barb1*^ and *Ren* ^*barb3*^ also contribute RN resistance to BARBREN-713 [24, 25]. In addition, BARBREN-713 is also homozygous for SSR markers CIR316_202 and BNL1231_197, which flank the *rkn-1* locus for RKN resistance [24, 26].

In this study, a global gene expression analysis was conducted using root RNA-sequencing data obtained from different genotypes of *G*. *hirsutum* with and without RN infestation. BARBREN-713 was selected as the resistant genotype, LONREN-1 was selected as the hypersensitive genotype, and two genotypes, DP90 and SG747, were pooled together and used as RN susceptible genotypes.

The aim of this study was to identify comparative gene expression responses from the RN susceptible, resistant, and hypersensitive genotypes, and to identify the important regulatory gene candidates located close to the RN resistance QTLs. The results presented in this study will extend the current understanding of RN resistance mechanism in cotton.

## Results and Discussion

**Fig 1:**
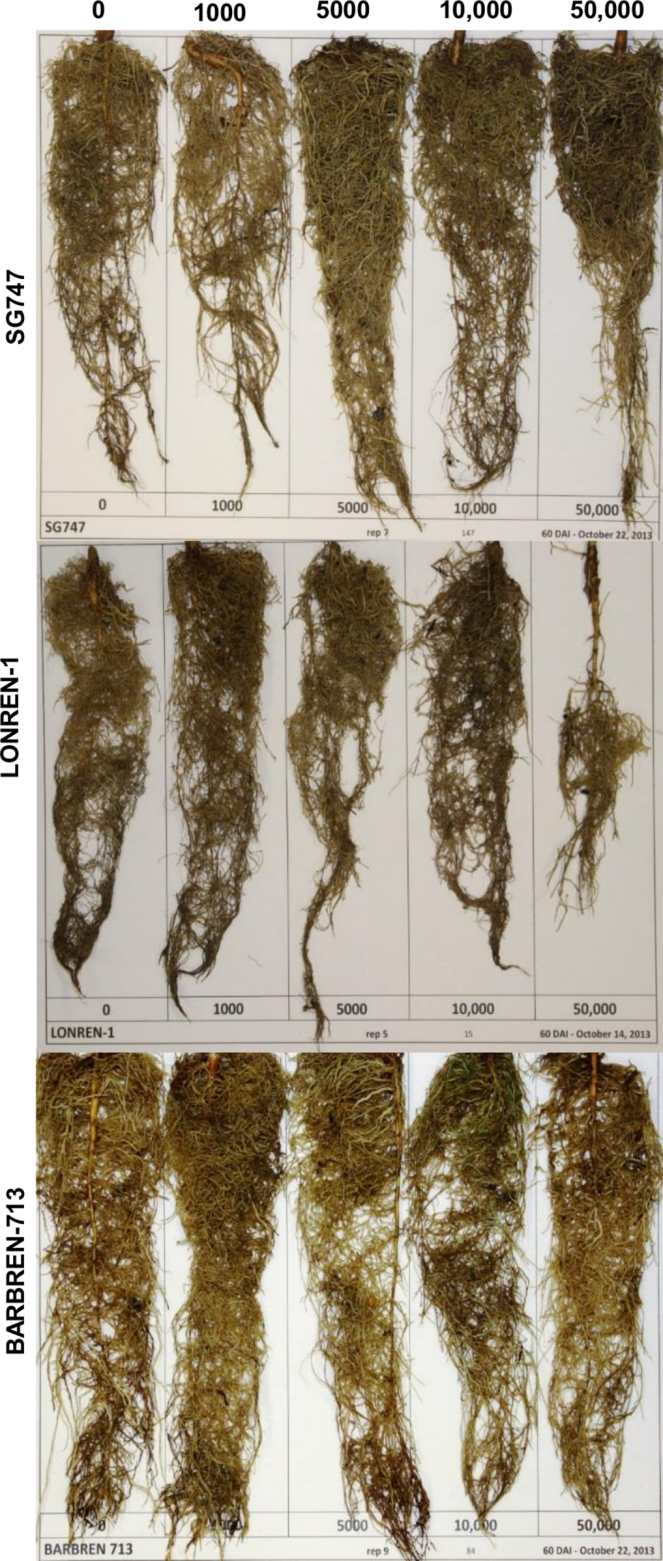
Reniform nematode susceptible SG747, resistant BARBREN-713, and hypersensitive LONREN-1 seedlings at various inoculum levels. Reniform nematode susceptible SG747, resistant BARBREN-713, and hypersensitive LONREN-1 seedlings at various reniform nematode inoculum levels. Numbers indicate *R*. *reniformis* number per 150 ml soil. Plants were photographed 60 days post reniform nematode infestation. Note the increased root biomass at higher inoculum levels for SG747, the relatively constant root biomass for BARBREN-713 at different inoculum levels, and the reduction in root volume at higher inoculum levels.

Upon RN infestation, different genotypes of cotton exhibited distinct root phenotypes following infestation with varying levels of RN [23]. A relative increase of root biomass was observed for susceptible genotypes under higher RN inoculum levels, a reduction of root volume was observed for hypersensitive genotype LONREN-1 with increased RN inoculum levels, while the root mass of resistant genotype BARBREN-713 remained constant at different RN inoculum levels (Fig 1).

### Sequencing and transcriptome assembly results

To obtain a global view of gene expression in different genotypes of cotton infested with RN, six paired-end (100bp) cDNA libraries were generated from cotton roots. These included: two libraries from pooled susceptible genotypes DP90 & SG747, either without RN infestation (DSU), or with RN infestation (DSI); two libraries from the hypersensitive genotype LONREN-1, either without RN infestation (L1U), or with RN infestation (L1I); and two libraries from resistant genotype BARBREN-713, either without RN infestation (B713U), or with RN infestation (B713I) (see S1 Fig).

Over 150 million raw reads were generated from all libraries (Table 1). After adaptor trimming and removal of low quality reads and reads shorter than 30bp, over 93 million reads (61% of the total raw reads) were obtained (Table 1).

**Table 1:** Summary statistics of sequenced reads from each library.

Libraries	DSU	DSI	L1U	L1I	B713U	B713I	Total
**Raw reads**	19,778,108	18,687,950	14,897,934	14,136,410	42,536,320	42,906,218	152,942,940
**HQR** ^**1**^	11,118,360	11,928,236	10,329,006	8,258,406	26,240,970	25,504,478	93,379,456
**(% Raw reads)**	(56.2%)	(63.8%)	(69.3%)	(58.4%)	(61.7%)	(59.4%)	(61.0%)

^1,^ HQR: High quality reads (Raw reads were trimmed in CLC genomic workbench with the following parameters after adapter trimming: ambiguous trim = yes; minimum number of nucleotide in reads = 30; quality limit = 0.05)

**Fig 2:**
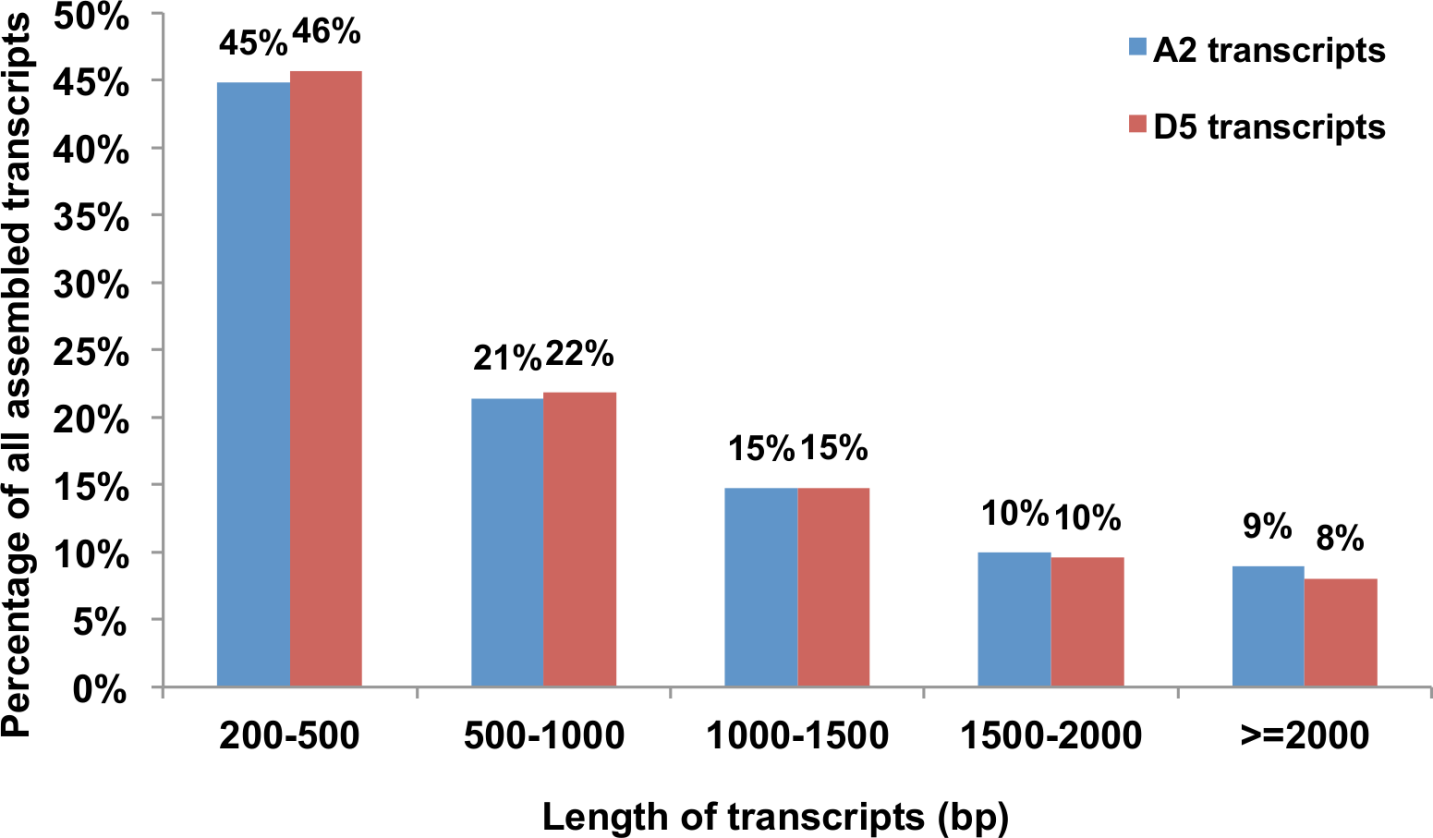
Length distribution of assembled transcripts. The percentage of total transcripts in various length classes is shown for A2 transcripts (blue), assembled with *G*. *arboreum* genome sequences as references; or D5 transcripts (red), assembled with *G*. *raimondii* genome sequences as references.

These paired-end sequence reads from all samples were pooled together to construct two sets of reference transcriptome assemblies using *G*. *arboreum* and *G*. *raimondii* genome sequences as references (Table 2) (see methods for details). The assembly derived from the A2 (*G*. *arboreum*) genome contained 84,711 transcripts and the assembly derived from the D5 (*G*. *raimondii*) genome contained 80,353 transcripts. These two assemblies were used as a *G*. *hirsutum* root reference transcriptome for all subsequent analyses (Table 2). Notably, A2 and D5 transcripts exhibited similar assembly statistics (Table 2) and length distributions, with ~45% transcripts between 100-500bp and ~55% transcripts greater than 500bp (Fig 2). In addition, a similar number and percentage of high quality reads from each library aligned back to the assembled A2 and D5 transcripts (Table 3).

**Table 2:** Summary of assembly results.

	A2 transcripts^1^	D5 transcripts^2^
**Total transcripts**	84,711	80,353
**N50 (bp)**	1,405	1,347
**Average length (bp)**	876	849
**Min length (bp)**	201	201
**Max length (bp)**	6,118	5,776

^1^ Transcripts assembled with *G*. *arboreum* genome sequences as references.

^2^ Transcripts assembled with *G*. *raimondii* genome sequences as references.

**Table 3:** Assessment of read content of the assembly.

Libraries	DSU	DSI	L1U	L1I	B713U	B713I	Total
**A2 transcript mapped (% of HQR)**	8,177,704 (73.55%)	8,164,194 (68.44%)	5,446,051 (52.73%)	5,734,348 (69.44%)	15,678,556 (59.75%)	15,356,629 (60.21%)	58,557,482 (62.71%)
**D5 transcript mapped (% HQR)**	8,009,309 (72.04%)	8,071,604 (67.67%)	5,376,659 (52.05%)	5,602,212 (67.84%)	15,563,133 (59.31%)	15,248,999 (59.79%)	57,871,916 (61.97%)

Number and percentage of HQR (highly quality reads, see Table 1) from each library mapped to A2 or D5 transcripts assembly.

**Fig 3:**
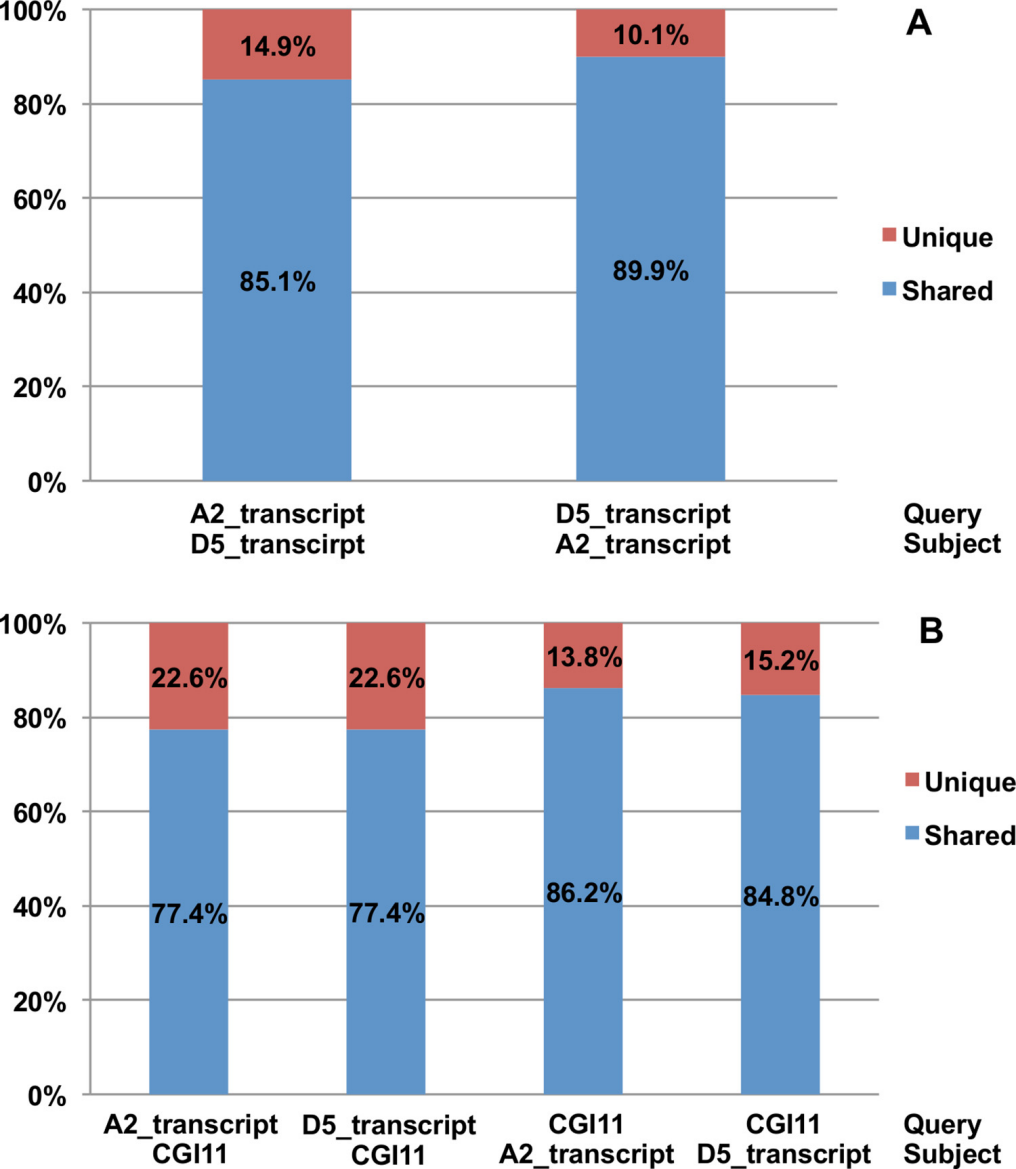
Sequence comparisons between assembled transcripts with public cotton transcript datasets. BLASTN searches were used to test the conservation and divergence between (A) the A2-derived and the D5-derived transcripts and (B) the A2-derived transcripts, the D5-derived transcripts, and the EST collections in Cotton Gene Index 11 (CGI11). An e-value < 10^−6^ was used as the criteria to identify the genes shared between each comparison.

To test the conservation and divergence between the A2-derived and the D5-derived transcripts, BLASTN searches were used to determine the percentage of apparently shared transcripts between the two libraries. Of the assembled transcripts obtained from the A2 genome and D5 genome, 85.1% and 89.9% respectively were shared between the two assemblies (Fig 3A). This result along with the fact that an average of about 60% of the high quality reads mapped to either A2 or D5 genome sequences is consistent with there being substantial conservation between the expressed sequences putatively derived from the A- and D-subgenomes of *G*. *hirsutum*. Sequences of the A2-derived transcripts, the D5-derived transcripts, and the EST assemblies in Cotton Gene Index 11 (CGI11, http://compbio.dfci.harvard.edu/tgi/cgi-bin/tgi/gimain.pl?gudb=cotton) were also compared to examine the transcriptome coverage and novelty of the newly assembled transcripts using the method described by [27]. Approximately 85% of CGI11 EST sequences were homologous to the A2- and D5- subgenomes-derived transcripts. That is, many known cotton ESTs are represented in the self-assembled root transcripts (Fig 3B). Using the reverse query, 22.6% of both A2 and D5 transcripts were unique, not matching any sequences in CGI11 (Fig 3B). Thus, while the newly assembled root transcripts have good depth of coverage of the known ESTs, they also contain unique assembled ESTs not found in existing EST collections making them useful as references for downstream analysis. These seemingly unique sequences likely relate to the fact that our databases were derived from root tissues, and thus, a significant number of root-derived EST sequences are revealed here.

### *R*. *reniformis*-responsive genes in *G*. *hirsutum* roots

To determine how many genes were differentially expressed in the three genotypes and whether there was variation before and after RN infestation, the number of expressed transcripts as reflected by the number of reads per kilobase of transcript per million mapped reads (RPKM) for each library is shown in S1 Table. Out of the 165,064 A2-derived and D5-derived transcripts, approximately 50% were expressed in each library with RPKM ≥ 2, and ~30% of the transcripts had expression values of RPKM ≥ 5 (S1 Table). In general, similar numbers of genes were expressed in different genotypes, and RN infestation did not seem to have an effect on the number of expressed genes.

**Fig 4:**
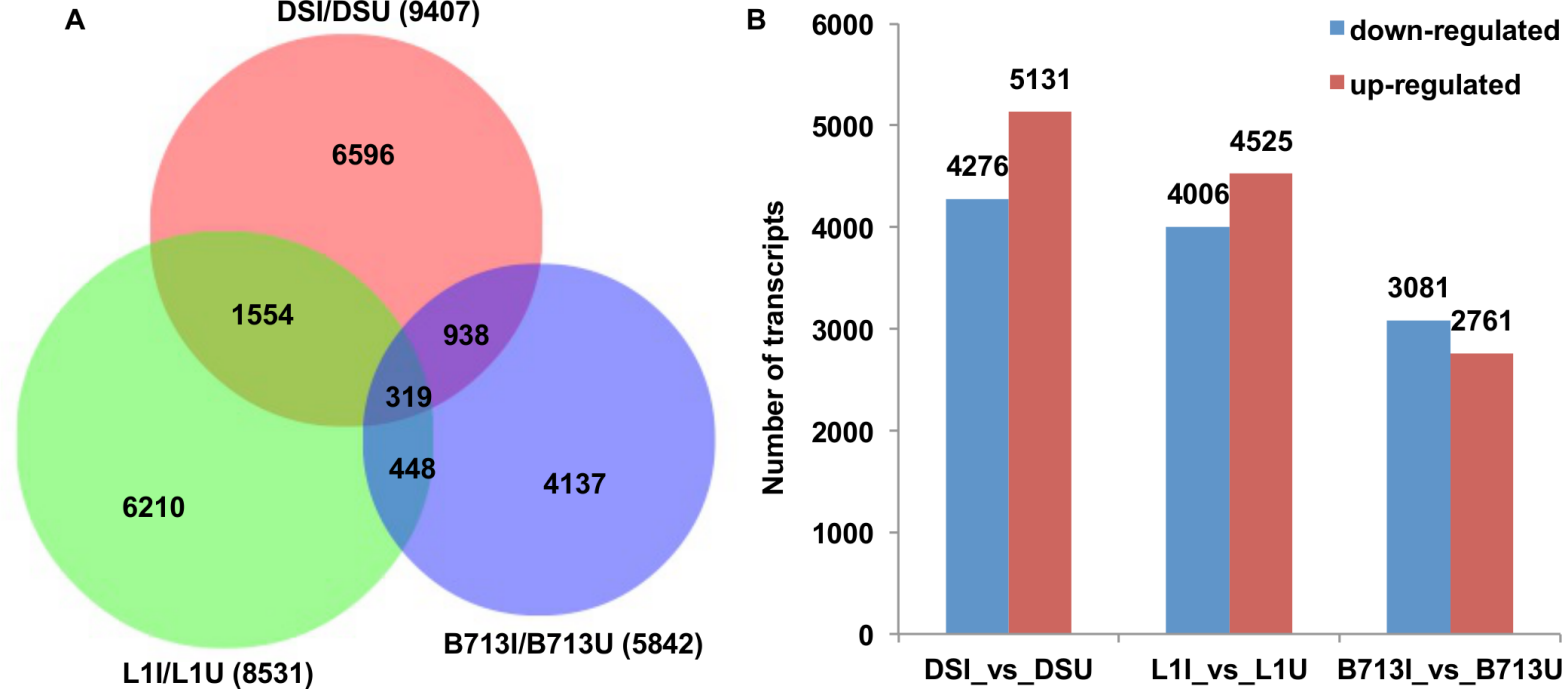
Number of *R*. *reniformis*-responsive transcripts. (A) Venn diagram shows the number of *R*. *reniformis*-responsive transcripts in each genotype; (B) The number of up- and down- regulated transcripts in each genotype in response to *R*. *reniformis* infestation.

In order to determine the regulation of cotton root transcripts in response to RN infestation, differential expression analysis (see methods for details) was performed between RN uninfested and infested libraries for each genotype. As a result, 9,407 transcripts were RN responsive in DS, 8,531 in L1, and 5,842 in B713 (Fig 4A). There were 1,873 RN-responsive transcripts common between DS and L1, 767 between L1 and B713, and 1,257 between DS and B713 (Fig 4A). Each of these results included the 319 transcripts that were differentially expressed in all three genotypes after RN infestation (Fig 4A). Thus, compared to the number of RN-responsive transcripts found for each genotype, a small number of transcripts were shared among the three genotypes. This might because: (1) the assembled transcripts (including isoforms of a single unigene) instead of unigenes were used for analysis, and different isoforms of the same gene were regulated differently; (2) homeologous genes from the A-subgenome and D-subgenome with similar biological functions were differentially regulated in the three genotypes; (3) genotypic variance between the three genotypes resulted in the sequence variance (e.g. SNPs) of the RN-responsive transcripts from each genotype; (4) because of the lack of replication, only partial transcripts were identified as RN-responsive making the clarity of the result difficult to ascertain, and/or (5) only a subset of shared differentially expressed transcripts were identified [28]. Some evidence supporting each of these explanations can be ascertained by inspection of the results, but no single cause appears to be responsible for this result.

Fig 4B shows that slightly more putative transcripts were up-regulated rather than down-regulated in DS and L1 after RN infestation, while slightly more transcripts were down-regulated rather than up-regulated in B713 after RN infestation.

**Fig 5:**
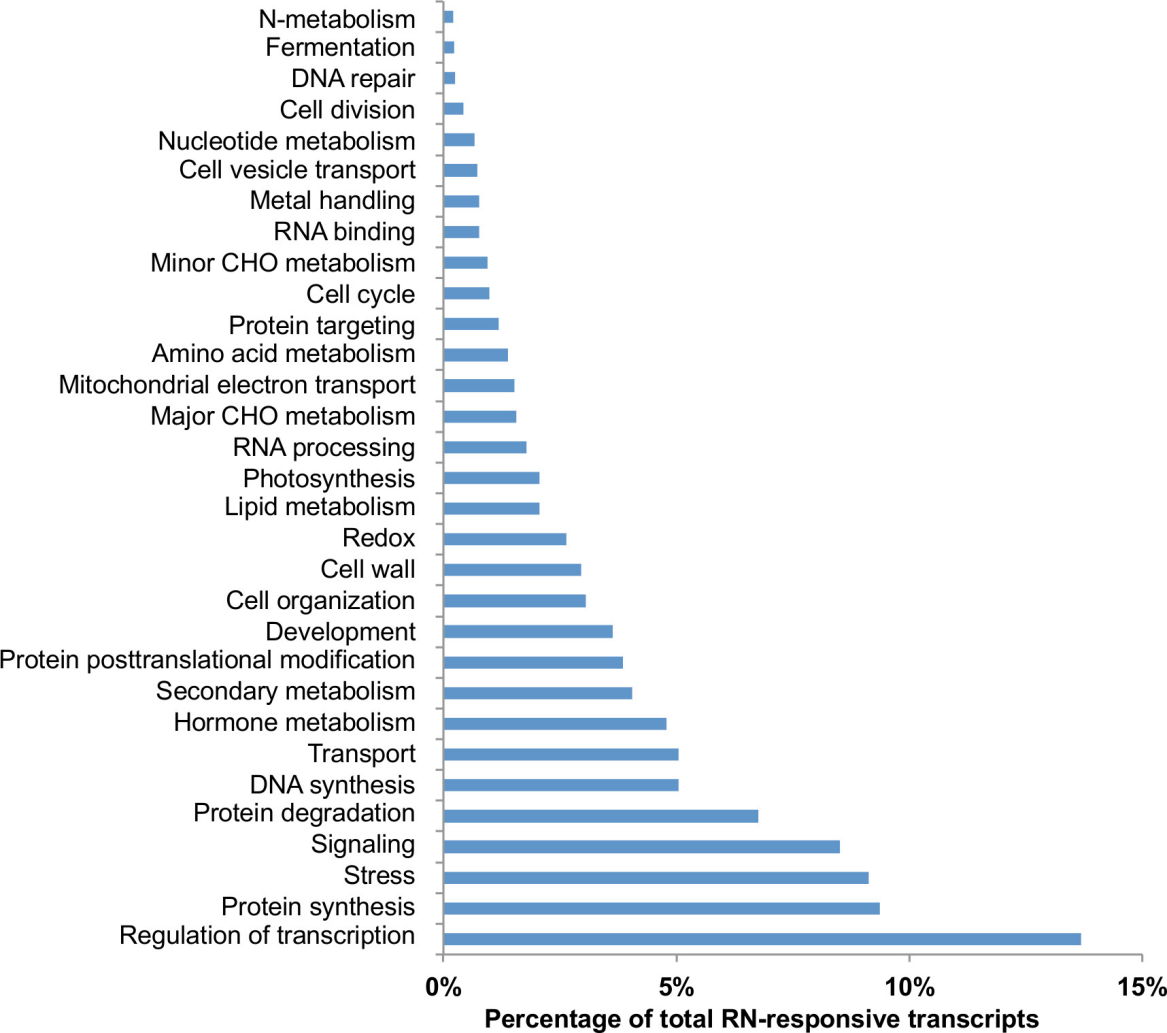
Gene Ontology categories of *R*. *reniformis*-responsive transcripts. The percentage of *R*. *reniformis*-responsive transcripts in each biological pathway category is shown. A transcript was considered *R*. *reniformis*-responsive if the FDR corrected P-value is smaller than 0.01 and the fold change value is more than 2.

Following differential expression analysis, all RN responsive transcripts were annotated using BLASTX identifying corresponding sequences in the *G*. *raimondii* and *G*. *arboreum* gene models. Based on the gene ontology term assigned for each RN-responsive transcript, the transcripts could be grouped into gene ontology (GO) categories (Fig 5). The expression of transcripts involved in transcriptional regulation, stress response, hormone metabolism and signaling, secondary metabolism, cell wall biosynthesis and degradation, and redox reactions were among the categories of genes known to have significant involvement in generic plant nematode interactions [14, 29]. Genes classified into other GO categories were also differentially regulated during cotton responses to RN infestation, including protein synthesis, signaling, protein degradation, DNA synthesis, transport, protein posttranslational modification, development, and cell organization. Among them, the signaling category contains many *R-genes* that were differentially regulated between genotypes without RN infestation.

#### Cell wall

As the initial physical barrier to protect plants from pathogen attack, partial dissolution of the plant cell wall matrix is required for the successful progression of nematode parasitism [30]. Several plant genes involved in cell wall biosynthesis and modification have demonstrated dynamic regulation during RKN and/or CN infestation [31–33].

A total of 137 RN responsive transcripts encoding cell wall-related enzymes were differentially expressed in at least one genotype, including 90 transcripts involved in cell wall loosening and/or degradation, and 47 transcripts involved in cell wall synthesis (Table 4). Among these RN responsive transcripts, most transcripts involved in cell wall loosening and degradation were up-regulated in susceptible genotype DS and down-regulated in hypersensitive genotype L1 after RN infestation. While fewer transcripts were differentially expressed (Fold change value > 2 & FDR P-value < 0.01) in B713I compared to B713U, relatively more were down-regulated (Table 4). These changes are consistent with the apparent RN responsive phenotype of each of the genotypes [29]. Moreover, the stimulation of more expansin and cellulose synthase transcripts in susceptible genotype DS (Table 4) may be necessary for cell wall relaxation during the formation of nematode induced root syncytia [29, 31, 34].

**Table 4:** Categories and numbers of RN-responsive transcripts involved in cell wall biosynthesis and degradation in cotton root tissues.

	DSI vs. DSU		L1I vs. L1U		B713I vs. B713U		
Cell Wall related pathways	Up	Down	Up	Down	Up	Down	Total
**Cell wall loosening & degradation**							**90**
Xyloglucan endotransglucosylase	28	0	0	22	1	0	35
Pectinase	13	6	6	5	1	5	31
Expansin	7	4	1	11	1	2	18
Mannan-xylose-arabinose-fucose	0	0	0	3	0	1	4
Cellulases and beta -1,4-glucanases	0	0	0	0	2	0	2
**Cell wall synthesis**							**47**
Cellulose synthesis	13	3	5	8	3	7	30
Cell wall precursor synthesis	6	0	1	8	0	3	15
Hemicellulose synthesis	0	2	0	0	0	0	2

In addition to being a passive physical barrier, plant cell walls can also monitor self-integrity, however no regulatory signaling molecules or pathways have been elucidated to date [35]. While studies have linked enhanced plant resistance to specific pathogens including bacteria, fungi, and aphids with reduced cellulose production [36, 37], the inhibition of cell wall synthesis-related transcripts in L1I and B713I (Table 4) appears consistent with their higher resistance levels to RN.

#### Hormone metabolism and signaling

Phytohormones are important players in both *R*-gene mediated resistance and plant basal defense responses to invading nematodes [14]. They either regulate plant responses to nematodes through pathogenesis related (*PR*) proteins and/or other resistance related factors, or affect nematode parasitism through manipulation of nematode feeding site initiation and development [14].

In this study, 111 transcripts involved in hormone synthesis and signaling pathways were found to be RN responsive in at least one genotype. This includes 57 transcripts connected to auxin (AX) pathway, 5 to cytokinin (CK), 16 to jasmonic acid (JA) pathway, 9 to ethylene (ET) pathway, 3 to salicylic acid (SA), 4 to abscisic acid (ABA), and 17 to gibberellic acid (GA) (Table 5).

**Table 5:** Categories and numbers of *R*. *reniformis*-responsive transcripts involved in hormone metabolism and signaling in cotton root tissues.

Hormone related pathway	DSI vs. DSU		L1I vs. L1U		B713I vs. B713U		
	Up	Down	Up	Down	Up	Down	Total
**Auxin**							**57**
AX_export	9	0	0	1	0	0	9
AX_response_ARF	0	1	0	2	0	1	3
AX_response_SAUR	0	7	9	9	2	1	25
AX_response_AUX/IAA	2	3	1	5	1	1	12
AX_response_GH3	4	0	2	3	0	1	8
**Cytokinin**							**5**
CK_biogenesis	0	0	0	2	0	0	2
CK_degradation	0	0	0	1	0	0	1
CK_activation	0	1	1	0	0	0	2
**Jasmonic acid**							**16**
JA_biogenesis	2	2	2	6	2	1	11
JA_responsive	4	0	0	0	0	0	4
JA_metabolic	1	0	0	0	0	0	1
**Ethylene**							**9**
ET_biogenesis	4	0	0	3	1	1	9
**Salicylic acid**							**3**
SA_metabolic	0	0	2	1	0	0	3
**ABA**							**4**
ABA_response	1	1	0	2	0	1	3
ABA_biogenesis	0	0	0	1	0	0	1
**Gibberellic acid**							**17**
GA_response	1	6	2	10	1	1	17

It should be noted that all 9 AX export related transcripts were up-regulated in DSI compared to DSU, 1 was repressed in L1I compared to L1U, and none of them were significantly differentially expressed in B713I compared to B713U (Table 5). Local and transient accumulation of AX in nematode feeding cells has a supportive role in nematode feeding site establishment and development [38–43]. Thus, data from this study extend the positive role of the up-regulated AX export-related transcripts to RN induced syncytium development in cotton roots.

Transcripts involved in AX responses were also differentially expressed (Table 5). Specifically, *ARF9* and *ARF19* orthologs were strongly repressed in L1I and B713I compared to their RN uninfested counterparts, and an *ARF8* ortholog was greatly down-regulated in DSI (Table 6). Correspondingly, in Arabidopsis-beet cyst nematode compatible interactions, *ARF9* and *ARF19* were strongly induced in the syncytium, and neighboring cells at the early stage of syncytium development (2–3 days post infestation), whereas *ARF8* had limited expression in beet cyst nematode infected roots throughout the time observed (1–10 days post infestation) [43]. These previous findings together with our results strengthen the hypothesis that *ARF9* and *ARF19* may play a positive role in nematode feeding site development while *ARF8* may play a negative role.

**Table 6:** Hormone metabolism and signaling-related genes expression profile in response to *R*. *reniformis* infestation.

ID	Expression value (RPKM)							
	DSU	DSI	L1U	L1I	B713U	B713I	Gene model	Annotation
D5 GG23405|c2 g1 i1	0.1	0.1	25.2	0.1↓	32.4	0.1↓	Gorai.007G026900.1	ARF 9
D5 GG26293|c1 g1 i1	0.1	8.2	27.4	0.1↓	8.7	0.1	Gorai.001G017000.2	ARF 19
D5 GG365|c1 g1 i1	32.3	0.1↓	0.1	0.1	0.1	6.9	Gorai.006G008700.1	ARF 8
D5 GG21297|c1 g1 i1	0.1	45.7↑	0.1	0.1	0.1	0.1	Gorai.009G330500.3	JAZ 3
D5 GG10735|c1 g1 i4	15.5	38.8↑	15.5	14.2	4.9	5.2	Gorai.011G062000.1	JAZ 6
D5 GG12672|c0 g1 i3	11.1	48.7↑	11.2	17.5	0.7	0.1	Gorai.002G021800.1	JAZ 10
D5 GG12672|c0 g1 i2	23.5	47.5↑	20.6	5.7	6.3	4.1	Gorai.002G021800.1	JAZ 10
D5 GG4054|c0 g1 i1	0.1	10.1↑	0.1	0.1	0.1	0.1	Gorai.005G245200.1	JMT
D5 GG11052|c0 g1 i2	23.1	35.2	52.6	21.9↓	7.7	10.8	Gorai.011G100800.1	SAMT
D5 GG8133|c0 g1 i1	7.0	1.4	5.3	26.8↑	9.1	6.6	Gorai.013G029200.1	SAMT
D5 GG8133|c0 g1 i2	6.6	3.8	2.0	22.4↑	7.0	6.6	Gorai.013G029200.1	SAMT
D5 GG1339|c0 g1 i1	0.1	0.1	12.9	0.1↓	0.1	0.1	Gorai.006G118300.2	AO 2
D5 GG20031|c3 g1 i1	14.3	3.9	21.3	3.9↓	27.5	6.2↓	Gorai.009G275700.1	ABF 2
D5 GG5776|c2 g2 i1	0.1	14.8↑	0.1	0.1	0.1	0.1	Gorai.005G212400.1	GRAM
D5 GG26414|c1 g1 i1	57.8	12.5↓	139.0	46.9↓	28.9	12.8	Gorai.001G057000.1	HVA22

(↑) indicates the significant up-regulation of each transcript in a specific reniform nematode infested library compared to the corresponding uninfested library, and (↓) indicates the significant down-regulation of a transcript in a specific reniform nematode infested library compared to the corresponding uninfested library. Significant = (Fold change value 2 and FDR P-value < 0.01)

Most JA biogenesis-related transcripts in this study were down-regulated in L1I compared to L1U, though no obvious expression trend can be concluded for DSI vs. DSU or B713I vs. DSU (Table 5). JA-responsive genes (*JAZ*) are transcriptionally up-regulated by JA and are repressors of JA signaling by inhibiting transcription factors that regulate early JA-responsive genes [44]. In this study, four *JAZ* orthologs were RN responsive. All of these appeared to be induced in susceptible genotypes (DS) after RN infestation, whereas no significant differential expression was detected in L1I or B713I (Table 6). One *JMT* ortholog, a stress responsive gene functioning in generation of MeJA by JA methylation [45], also exhibited significant up-regulation in response to RN infestation in the DS genotype (Table 6).

Direct application of JA induces resistance responses to RKN in tomato [46] in a dose dependent manner [47], and JA was found to be an indispensable signal in rice resistance to RKN [48]. On the other hand, JA signaling via COI receptor appears to be required for susceptibility to RKN [49]. Similarly, result from other studies inferred the positive role of JA biosynthesis in plant susceptibility to RKN [50, 51]. While JA serves as a significant player in plant defense responses to CN [52] and herbivory-induced wounding [53,54], our data are more supportive of a positive role of *JAZ* and *JMT* orthologs in plant susceptibility to RN, although no specific role of JA accumulation in cotton responses to RN can be inferred. Hence, it appears that the effect of JA may differ in response to different species of parasitic nematodes.

As for the SA pathway, 3 orthologs of *SAMT* were found to be RN responsive in this study (Table 6). *SAMT* modulates SA levels by converting SA to methyl-SA, and methyl SA can function as a mobile signal, mediating systemic acquired resistance in some plants [55]. In soybean plants specifically overexpressing *SAMT* confers resistance to soybean cyst nematode has been demonstrated [56]. Moreover, SA was proposed to form a self-amplifying feedback loop with ROS (i.e. H_2_O_2_) in potentiating plant HR [57]. In our studies, the specific differential expression of *SAMT* orthologs in L1I is consistent with *SAMT* playing a role in RN resistance and hypersensitive cell death responses.

ABA functions as a widespread growth inhibitor, inhibiting cell division and cell expansion, but promoting cell differentiation [58]. The AO2 protein catalyzes the last step of ABA synthesis, and the *ABF2* gene encodes a bZIP-type transcription factor that regulates downstream ABA-induced gene expression [58]. The ABA responsive GRAM domain-containing proteins produced from the ABA-responsive-1 (ABA1) and HVA22 genes have been associated with hypersensitive cell death [59, 60]. This study revealed that orthologs of the above four genes may be RN responsive (Table 6), thus linking ABA synthesis and signaling with RN responses in cotton roots.

#### Redox, secondary metabolism, pathogenesis, and other stress

Global gene expression analysis identified highly represented RN responsive transcripts involved in plant redox, metabolism (143 transcripts), secondary metabolism (220 transcripts), and pathogenesis-related proteins (33 transcripts) (Table 7). In addition, over 70 transcripts annotated as Heat Shock Proteins (HSP) and more than 50 transcripts annotated as nodulin-like enzymes were RN responsive in at least one genotype (Table 7). Several transcripts categorized as HR-related, growth regulating factor, and sRNA biogenesis also exhibited statistical differential expression in response to RN infestation (Table 7). While the induction of nodulin-like and HR-related genes in DSI vs. DSU and L1I vs. L1U (Table 7) are consistent with their roles in assisting increased nutrient transport [61] to syncytium in RN susceptible responses and triggering HR-like responses respectively, dynamic regulation of HSP and growth regulating factor coding genes by sRNA have been implicated in plant susceptibility and/or resistance to CN [62, 63].

**Table 7:** Categories and numbers of *R*. *reniformis*-responsive transcripts involved in stress response-related pathways.

	DSI vs. DSU		L1I vs. L1U		B713I vs. B713U		
Pathways	Up	Down	Up	Down	Up	Down	Total
**Redox**							**143**
peroxidase	34	7	16	35	1	2	79
ascorbate peroxidase	2	0	0	2	0	0	4
dismutases and catalases	1	2	1	2	2	0	8
thioredoxin	3	17	14	6	2	3	35
glutaredoxins	0	6	5	6	2	0	17
**Secondary metabolism**							**220**
isoprenoids metabolism	14	34	30	13	3	8	81
phenylpropanoids metabolism	41	23	33	22	11	8	111
dirigent like	6	11	15	0	1	1	28
**Pathogenesis related**							**33**
chitinase	6	0	6	6	3	1	20
Thaumatin like	2	3	1	2	6	1	13
**Other**							**135**
HSP20	4	23	1	41	2	1	52
HSP40	2	1	2	5	3	0	11
HSP70	0	2	0	3	1	2	4
HSP90	3	0	0	0	0	2	3
Nodulin like	22	6	9	15	2	12	57
HR-related	0	0	2	0	0	0	2
Growth regulating factor	1	1	0	0	0	0	2
sRNA biogenesis	1	0	0	3	0	0	4

One hundred forty three transcripts involved in plant redox reactions were RN responsive (Table 7). Among them, most transcripts annotated as peroxidase were up-regulated in DSI vs. DSU, down-regulated in L1I vs. L1U, but not statistically differentially regulated in B713I vs. B713U (Table 7). There are three main classes of plant peroxidases, of which apoplastic localized class III peroxidases can either act as H_2_O_2_ scavengers or generate H_2_O_2_, depending on the specific physiological conditions [64]. Although the specific classes of these 79 peroxidases are unclear, the possibility that they were involved in cell death in DS especially L1 (Fig 1) [23] cannot be excluded. Specifically, different peroxidase genes appear to play distinct roles in plant responses to cereal cyst nematode infection [65]. In plant responses to RKN infection, the repression of *TPX1*, a peroxidase gene involved in cell wall lignification, was demonstrated to hinder nematode feeding site expansion and RKN parasitism [12]. Hence, further additional experimental evidence is warranted to examine the specific roles of different peroxidases in plant responses to RN infestation.

Ascorbate peroxidase, superoxide dismutase (including Cu/Zn and Mn superoxide dismutase), and catalase belong to plant class I peroxidase family, and they serve as ROS scavengers, along with thioredoxin and glutaredoxin [66]. Notably, 35 thioredoxin annotated transcripts showed differential expression, with more transcripts down-regulated in DSI vs. DSU but up-regulated in L1I vs. L1U, in a reverse direction as compared to the expression patterns of transcripts annotated as peroxidase (Table 7). While HR type cell death has been observed in L1 under high level of RN infestation [23], the induction of transcripts annotated as ROS scavenger might be in part responsible for the HR [67], playing a positive role in L1 resistance against RN.

In addition to transcripts involved in redox reactions, 220 transcripts involved in secondary metabolism were RN responsive in at least one genotype (Table 7). The phenylpropanoid pathway, specifically, has a known role in plant defense against pathogens resulting from cell wall strengthening effects, making more stress hormone (i.e. SA), and serving as an antioxidant in ROS scavenging [68, 69]. Accordingly, in all three genotypes after RN infestation, the number of up-regulated transcripts involved in the phenylpropanoid pathway were greater than those that were down-regulated (Table 7).

Twenty eight transcripts annotated as dirigent-like proteins were RN responsive (Table 7). Dirigent proteins play roles in plant secondary metabolism especially the biosynthesis of lignin, which strengthens plant cell walls and helps plants defend against pathogens [70]. In cotton responses to RN, lignin deposition was a characteristic in plant HR responses resulting in reinforcement of the cell walls surrounding nematode infection sites; thus creating a barrier to inhibit the spread of the infection [71]. As such, all of the RN responsive dirigent-like protein transcripts were up-regulated in the L1 genotype after RN infestation (Table 7), which showed a hypertensive-type cell death response after RN infestation [23].

Chitinase and thaumatin-like proteins are both pathogenesis related (PR) proteins that were induced in response to pathogen infection and are often associated with the production of antimicrobial secondary metabolites [72]. Others have found that genes coding for both proteins were differentially expressed upon RKN or CN infection [7, 13, 73], and the thaumatin like *PR-5* gene has been well correlated with nematode resistance [5, 12, 74, 75]. In this study, transcripts annotated as chitinase were significantly up-regulated in DS, while thaumatin-like transcripts were up-regulated in B713 (Table 7). Taken together, *PR-5* gene appeared to be a positive factor in generic nematode resistance as well as in B713 genotype specific RN resistance.

#### Transcriptional regulation

Transcription factors are important regulators in plant responses to nematode infestation by inducing or suppressing defense-related genes [14]. Two hundred and ninety four transcripts annotated as transcription factors were RN responsive. This constitutes the most abundant category of genes that show differential expression in response to RN (Table 8). The most abundant RN responsive transcription factor families in different genotypes include MYB, ERF, WRKY, HSF, GRAS, BZIP, and NAC families. While different transcription factors in each family may have distinct roles in cotton responses to RN, all of the highly represented families (Table 8) are important regulators in either root development and/or plant immunity responses.

**Table 8:** Families and numbers of *R*. *reniformis*-responsive transcripts annotated as transcription factors.

	DSI vs. DSU		L1I vs. L1U		B713I vs. B713U		
Families	Up	Down	Up	Down	Up	Down	Total
MYB	19	5	7	13	3	6	48
ERF	13	6	6	10	6	5	44
WRKY	10	3	10	9	2	1	29
HSF	5	2	2	3	2	3	14
GRAS	6	2	4	1	0	2	11
BZIP	4	2	3	0	2	0	10
NAC	0	1	1	2	1	2	7

MYB family proteins are involved in the regulation of flavonoid biosynthesis, root hair patterning, and lateral root formation [76]. ERF transcription factor expression is regulated by plant hormones, including SA, JA, ET, CK, and ABA [77]. WRKYs are well known as key regulators in plant innate immunity [78], and different *WRKY* genes have been shown to regulate plant responses to nematode infestation [79–81]. HSF transcription factors regulate the expression of heat shock molecular chaperones [82]. SCARECROW transcription factor in GRAS protein family regulates root radial patterning [83]. TGA transcription factor in bZIP family contributes soybean cyst nematode resistance when overexpressed in soybean [74], and NACs are central components of plant innate immunity hormone signaling and ROS signaling [84]. MYC2 transcription factors in bHLH family are master regulators in JA signaling [85].

### Specific differences between three genotypes without *R*. *reniformis* infestation

**Fig 6:**
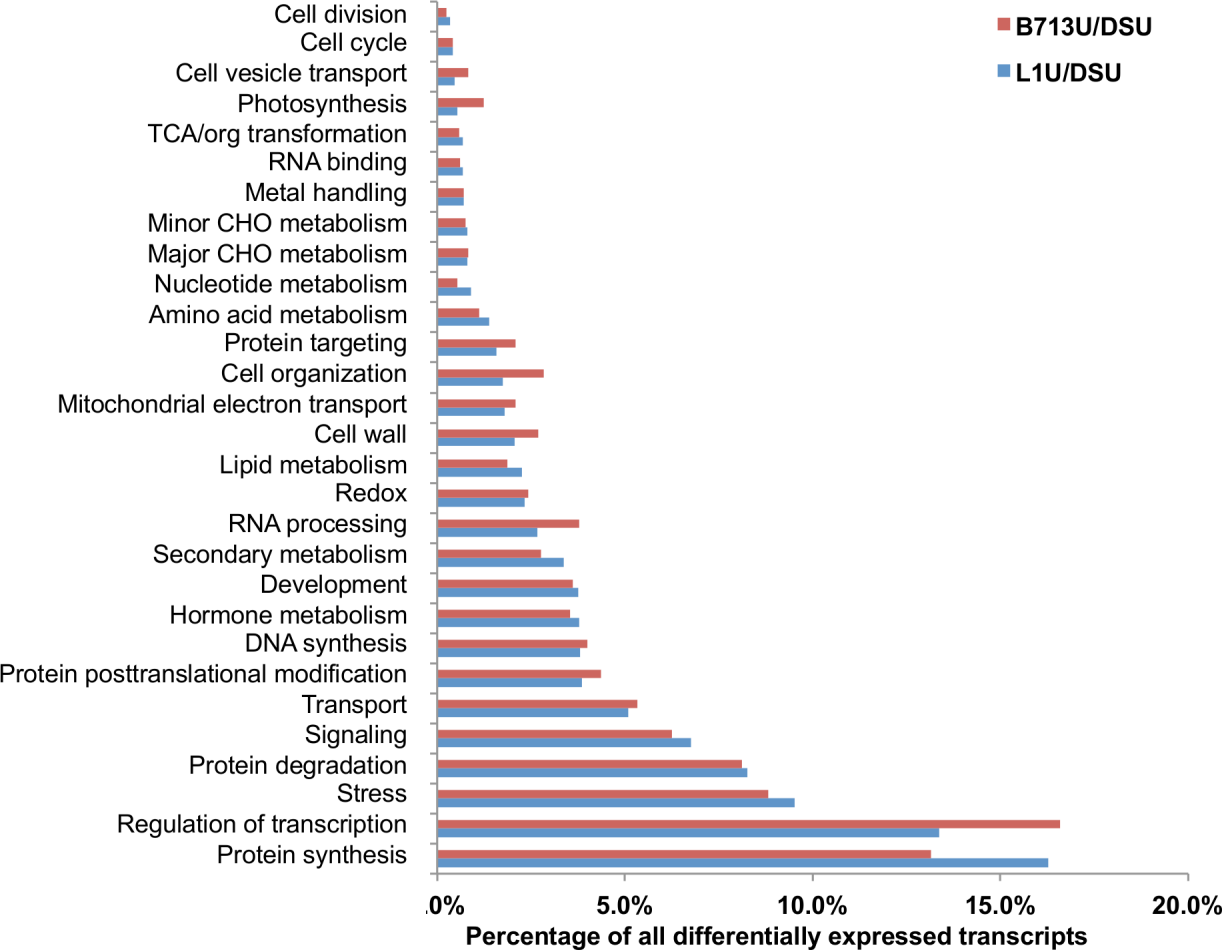
Gene ontology categories of differentially expressed transcripts from cotton root tissues comparing susceptible to hypersensitive or resistant genotypes without *R*. *reniformis* infestation. The percentage of differentially expressed transcripts between genotypes in each biological pathway category is shown. A transcript was considered to be differentially expressed if the FDR corrected P-value is smaller than 0.01 and the fold change value is more than 2.

To determine the constitutive genotypic differences between the three genotypes, expression analysis was also conducted among the susceptible, the hypersensitive, and the resistant genotypes without RN infestation. 4,171 and 8,503 transcripts demonstrated a significant (FDR corrected P-value < 0.01) increase in abundance in L1U and B713U, respectively, compared to DSU, whereas 5,984 and 4,709 transcripts demonstrated a significant (FDR corrected P-value < 0.01) decrease in abundance in L1U and B713U, respectively (S2 Fig). Similar sets of differentially regulated biological pathways were identified for L1U vs. DSU and B713U vs. DSU (Fig 6).

A greater percentage of differentially expressed transcripts was observed in the B713U to DSU comparison for the categories of transcription regulation, RNA processing and cell organization reflecting that a greater number of transcripts associated with gene regulation are differentially expressed, while in the L1U to DSU comparison only the category of protein synthesis demonstrated an larger increase in expression.

#### Association of differentially expressed transcripts to *R*. *reniformis* resistance QTLs

The sequences of six SSR markers (BNL3279, BNL4011, BNL1721, BNL569, BNL1551, and GH132), genetically linked to RN resistance QTLs [25, 86], were retrieved from the cotton EST database [87]. Five of these SSR marker sequences (all except BNL1721 that only mapped to the *G*. *raimondii* genome) mapped to both the *G*. *arboreum* [21] and the *G*. *raimondii* [19] genomes (Table 9).

**Table 9:** Mapping positions of RN resistance QTLs-associated SSR markers.

SSR	Sequence length (bp)	Chromosome	Start	End
BNL3279	619	A2 Chr10	74030524	74029915
BNL4011	337	A2 Chr4	133838455	133838116
BNL569	228	A2 Chr13	57120961	57121190
BNL1551	210	A2 Chr4	110408238	110408414
GH132	739	A2 Chr10	14211757	14211051
BNL3279	619	D5 Chr7	56142481	56143089
BNL4011	337	D5 Chr7	54550456	54551582
BNL1721	241	D5 Chr13	30666694	30666468
BNL569	228	D5 Chr13	47353483	47353704
BNL1551	210	D5 Chr7	49018320	49018506
GH132	739	D5 Chr7	53372611	53372033

A2: genome of *G*. *arboreum*; D5: genome of *G*. *raimondii*

To identify the transcripts that were differentially expressed among genotypes that were located near one of the RN-resistance QTL SSR markers, the differentially expressed transcripts (Fold change value > 2 & FDR P-value < 0.01) were mapped against the *G*. *arboreum* and *G*. *raimondii* genomes. The chromosome location of each of these transcript was noted and compared with those of SSR markers, to find the transcripts that mapped within 1 megabase of the different SSR markers, following the method described in [88].

**Fig 7:**
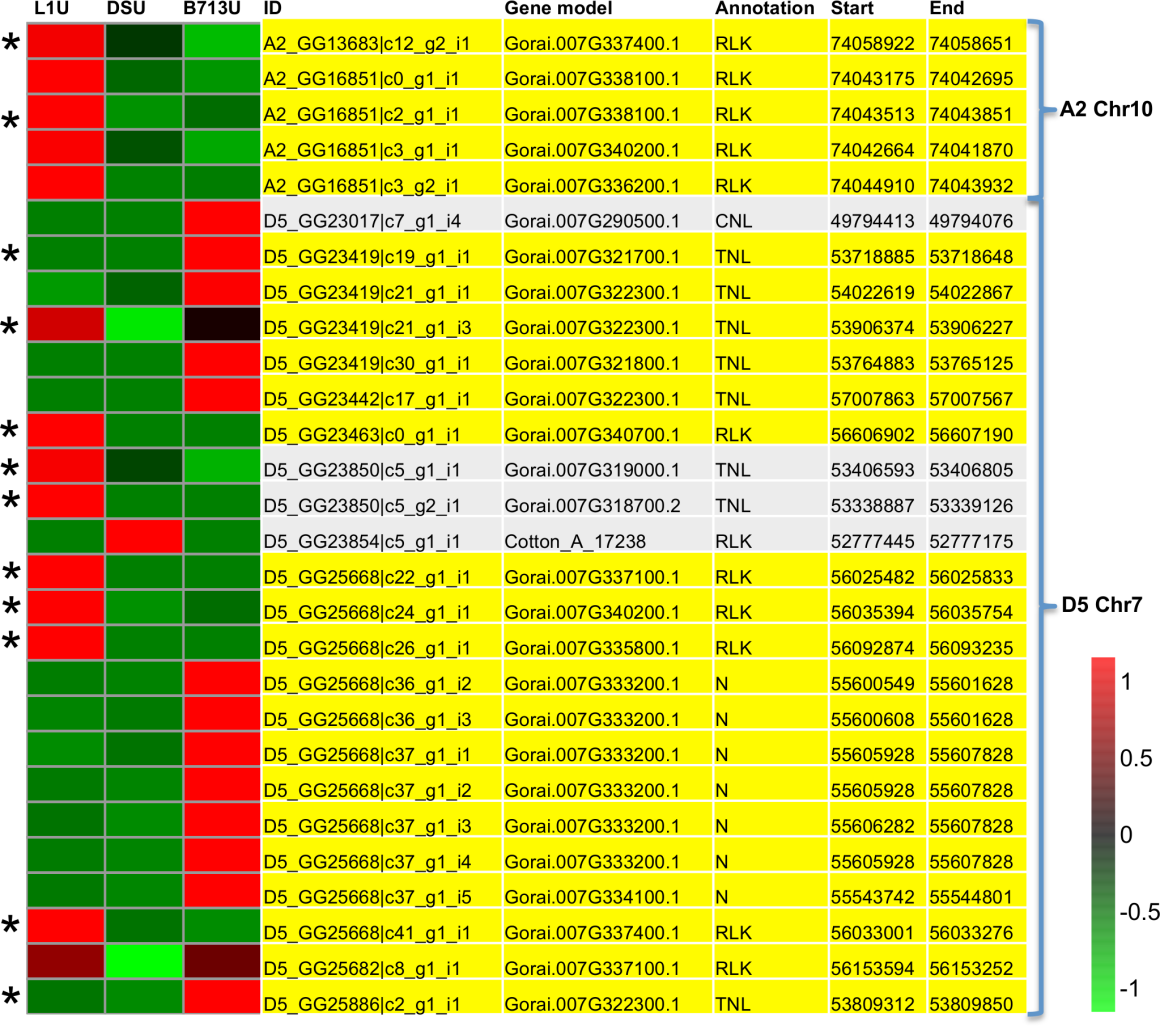
Expression of *R*. *reniformis* resistance-associated SSRs-adjacent *R*-gene annotated transcripts among the susceptible, hypersensitive, and resistant cotton genotypes. The expression of *R-*gene annotated transcripts that are within 1 megabase of *R*. *reniformis* resistance-associated SSR markers is shown. All but one transcript shows statistically significant higher expression in LONREN-1 (L1U) and/or BARBREN-713 (B713U) uninfested treatments than in susceptible genotype (DSU). Transcripts within 1 megabase of BNL3279 are highlighted in yellow, and transcripts that are also differentially expressed (Fold change value > 2 & FDR P-value < 0.01) in response to *R*. *reniformis* are denoted with asterisk. The colors represent the relative expression levels of each sequence in the three libraries examined, red indicates higher expression values, and green indicates lower expression values. RLK: leucine-rich repeat receptor kinase; TNL: TIR-NBS-LRR class disease resistance protein; CNL: CC-NBS-LRR class disease resistance protein; N: NB-ARC domain-containing resistance protein; A2: genome of *G*. *arboreum*; D5: genome of *G*. *raimondii*.

Notably, 28 transcripts annotated as *R*-genes were mapped within 1megabase of the different SSR markers, which accounted for 20% of the differentially expressed transcripts near SSR markers (S2 Table), and over 10% of all the differentially expressed *R-gene*-annotated transcripts (S3 Table). All but one of these *R*-genes had higher expression in L1U or B713U compared to DSU, and chromosome locations within 1 megabase of BNL3279 (highlighted in yellow) appear to be located in a “hot spot” where many of these differentially expressed *R-*gene-annotated transcripts cluster (Fig 7). SSR marker BNL3279 was associated with QTLs from both RN resistance resources: *G*. *longicalyx* and *G*. *barbadense* [25, 86]. Additionally, BNL3279 with the amplicon size of 132 bp was also in the flanking regions of *Ren* ^*ari*^, an RN resistance locus from *G*. *aridum* [89]. Identifying the important regulatory genes that are located in the BNL3279-linked QTL region may help us to understand the importance of BNL3279 in the cotton responses to RN.

*R*-gene encoded proteins, including those annotated as LRR receptor kinase, NBS-LRR class resistance proteins, and NB-ARC domain-containing resistance proteins, are classified into the “signaling” GO category. Both extra-cellular LRR domain containing R-receptor and intra-cellular R-receptor proteins (typically identified as NBS-LRR type R proteins) can sense invading pathogen-associated molecular patterns and mediate plant innate immunity signaling as well as various downstream defense responses [15].

In plant-nematode interactions, a series of *R*-genes have been cloned, that condition resistance to either RKN or CN, and most of these encode canonical intracellular NBS-LRR type R-receptor proteins [14]. While *R*-genes of this type have not been correlated with RN resistance to date, the fact that a large number of *R*-gene-annotated transcripts are located in the vicinity of the RN resistance QTLs and they were either up-regulated in L1U or B713U compared to DSU, makes possible the hypothesis that one or more of these mapped sequences could at least affect the different levels of RN resistance for the three genotypes tested. Particularly, 12 out of these 28 *R*-gene annotated transcripts were also differentially expressed in response to RN, with 4 of them induced and 1 repressed in DS, 7 and 2 repressed in L1 and B713 respectively (Fig 7; S4 Table). Two hundred and ninety transcripts annotated as *R*-gene in the “signaling” GO category were differentially expressed (Fold change value *>* 2 & FDR P-value < 0.01) in response to RN infestation in at least one genotype (S4 Table). Over 50% of these RN responsive *R*-gene annotated transcripts were located on chromosome 4 of *G*. *arboreum* and/or chromosome 7 of *G*. *raimondii*, where BNL3279, BNL4011, BNL1551, and GH132 were mapped (Panel A, S3 Fig), and most of them were clustered in the vicinity of the marker region on both chromosomes (Panels B and C, S3 Fig). Collectively, these data are consistent with the hypothesis that *R*-genes in the vicinity of the RN-resistance associated markers have a role in mediating different levels of resistance to RN, and their expression levels are dynamically regulated during responses to RN.

### Comparison of RNA sequencing results to quantitative real-time PCR analysis

To test the RNA-seq results, 10 differentially expressed transcripts (S5 Table) that were associated with specific RN responses, were selected for qRT-PCR. A comparison of the expression of each transcript by qRT-PCR and RNA-seq was made. Of the 10 transcripts, all but one receptor like kinase gene (A2_GG16851|c2_g1_i1) were detected, probably due to its low expression level. Of the 9 detected transcripts, the expression patterns of seven transcripts showed general agreement with RNA-seq results: the same trend of expression level change as determined by statistical analysis of RNA-seq data was observed. These seven transcripts include two HR-like lesion-inducing protein-related genes (A2_GG28654|c0_g1_i1 and D5_GG10821|c1_g1_i1), two receptor like kinase genes (D5_GG25668|c24_g1_i1 and D5_GG25668|c41_g1_i1), two NB-ARC domain-containing genes (D5_GG25450|c74_g1_i1 and D5_GG25672|c1_g1_i2), and one ACC-oxidase gene (D5_GG5758|c0_g1_i1) (S4 Fig). In contrast, the other two genes had qRT-PCR results that differed from RNA-seq results. They were one thaumatin-like gene (A2_GG25608|c1_g2_i1) and one auxin efflux carrier gene (D5_GG19325|c0_g1_i1) (S4 Fig). In addition, five out of the seven transcripts expression level change determined by qRT-PCR was statistically significant (P-value < 0.05) (S4 Fig). The discrepancy between RNA-seq and qRT-PCR results could have been caused by the use of different biological samples in the two separate experiments or could have been resulted from intrinsic differences in the manner of expression estimation made by the two techniques. In summary, the majority of the selected transcripts tested by qRT-PCR were generally expressed in the same manner as identified by the statistical analysis of RNA-seq data.

## Conclusions

Gene expression and metabolic studies have identified genes involved in various signaling pathways that regulate plant responses to RKN and/or CN. In the present study, RNA-sequencing was used to investigate global gene expression patterns in cotton susceptibility, hypersensitivity, and resistance to RN. The data presented indicate that genes involved in cell and cell wall architectures, hormone metabolism and signaling, ROS levels, cell death pathways, pathogenesis and genes involved in putative phytoalexin pathways were distinctly modulated between the RN susceptible, hypersensitive, and resistant genotypes. These results are consistent with cell wall metabolism being dynamically regulated in susceptible genotypes to support RN parasitism, and AX polar transport facilitating the formation of RN-induced syncytia in cotton roots. Compared to susceptible genotypes, genes involved in ROS production and scavenging are more significantly regulated in cotton resistance especially hypersensitive responses to RN. Specific families of genes might contribute to RN resistance without HR induction, such as thaumatin-like *PR-5* genes, and the formation of secondary metabolite may serve as a common factor in RN resistance.

By correlating the chromosomal positions of differentially expressed genes with RN QTL loci, 28 transcripts that were annotated as putative *R*-genes in the vicinity of RN-resistance-associated markers were identified that have a higher accumulation level in either the RN resistant genotype BARBREN-713 or the RN hypersensitive genotype LONREN-1 compared to the RN susceptible genotypes. *R*-genes that are in the vicinity of RN-resistance associated markers were also dynamically regulated in response to RN. Collectively, the data are consistent with an hypothesis that one or more *R-gene* receptors close to the RN resistance-associated SSR markers serve as critical molecular determinants of resistance to RN, and the dynamic regulation of their expression levels in response to RN infestation is critical in determining RN responses in cotton roots. Further experimental work is required to more specifically identify the important candidates among the list of possibilities.

In conclusion, several pathways and genes that could be considered in future functional analysis of different plant responses to RN have been identified from this transcriptome analysis. Further examination of the putative roles of these pathways and genes in RN susceptibility, hypersensitivity, and resistance is required, but a list of the most important candidate sequences was determined.

## Materials and Methods

### Plant material and stress treatment

Four genotypes of cotton were selected: two *R*. *reniformis* susceptible genotypes DP90 and SG747, one resistant genotype BARBREN-713, and another resistant genotype but with hypersensitive responses: LONREN-1. Cotton seedlings were infested with *R*. *reniformis* two weeks after planting and root samples were collected at 0, 1, 3, 5, and 10 days post infestation. Samples at 0 day were taken as uninfested controls, while samples from 1, 3, 5, and 10 days post-infestation were pooled and considered the *R*. *reniformis* infested samples (S1 Fig).

### cDNA library construction

Total RNA was extracted from root samples using the hot borate method [90]. Equal amount of root tissue from susceptible genotypes was combined as one susceptible control for RNA extraction, whereas root tissue from infested samples was equally combined for RNA preparation. After mRNA purification using GenElute mRNA miniprep kit (Sigma), six cDNA libraries were constructed using Mint cDNA synthesis kit (Evrogen). They are DP90 and SG747 uninfested cDNA library (DSU), DP90 and SG747 infested cDNA library (DSI), LONREN-1 uninfested cDNA library (L1U), LONREN-1 infested cDNA library (L1I), BARBREN-713 uninfested cDNA library (B713U), and BARBREN-713 infested cDNA library (B713I) (S1 Fig). The constructed libraries were sequenced on illumina 2000 HiSeq sequencer at the Genomics Core Facility at Emory University. Raw sequencing data is available for download at NCBI Sequence Read Archive under BioProject: PRJNA275155 (BioSamples: SAMN03381308; SAMN03381306; SAMN03381268; SAMN03381265; SAMN03381261; and SAMN03339739).

### Assembly

Before assembly, raw sequencing reads were trimmed by removing adaptor sequences, ambiguous nucleotides, low quality sequences, and short read length sequences (length below 30bp) with CLC Genomic workbench (version 5.5.1). The quality of raw reads and trimmed reads was checked by fastQC software (version 0.10.1). Given the large size of the data, a Trinity in silico normalization of the full data set was conducted before assembly, to reduce memory requirements and improve assembly runtime. Subsequently, the in silico normalized reads were aligned to the bowtie2 [91] built *G*. *raimondii* genome (version 2, [19]) reference and *G*. *arboreum* genome [21] reference with tophat2 [92] respectively. Both *G*. *raimondii* and *G*. *arboreum* genome guided assemblies of the normalized reads into transcripts and genes were carried out using Trinity with default parameters [93]. Transcripts assembled with *G*. *raimondii* and *G*. *arboreum* genome sequences as references were considered D5 and A2 subgenome sequences respectively. The pipeline used for transcriptome assembly is illustrated in S5 Fig.

### Expression analysis

As shown in (S5 Fig), to compute expression values of assembled transcripts in each library, the trimmed reads from each library were aligned to the combined set of transcripts using bowtie2, and RSEM [94] was executed to estimate expression values of every transcript based on the resulting alignments. The expression values of assembled transcripts in each library were presented in RPKM (reads per kilobase of transcript per million mapped reads).

Expression analysis and Kal’s statistical analysis were conducted in CLC genomic workbench (version 5.5.1). The calculated original P-values were additionally FDR corrected using CLC built-in method described in (Benjamini and Hochberg 1995). A transcript was considered to be differentially expressed if the FDR corrected P-value given by the above analysis was smaller than 0.01, and the fold change in RPKM normalized counts was more than 2. The online tool BioVenn [95] was used for the construction of Venn diagrams. The R statistical package was used for the construction of heat maps.

### Annotation and gene ontology categorization

For annotation of differentially expressed transcripts, the sequences were searched against the published *G*. *arboreum* [21] and *G*. *raimondii* [19] gene models using BLASTX (e-value <1e − 6) (S5 Fig). GO annotations of the published *G*. *arboreum* (http://cgp.genomics.org.cn/page/species/index.jsp) and *G*. *raimondii* (http://mapman.gabipd.org) gene models were used for the functional categorization of differentially expressed transcripts.

### Identification of potential *R*. *reniformis* resistance genes in the vicinity of resistance QTL

The sequences of six SSR markers (BNL3279, BNL4011, BNL1721, BNL569, BNL1551, and GH132), genetically linked to QTLs that are significantly associated with *R*. *reniformis* resistance [22, 24, 25, 86], were retrieved from the Cotton EST database [87]. To identify their position on the published *G*. *arboreum* and *G*. *raimondii* genomes, SSR marker sequences were BLASTN against the *G*. *raimondii* and *G*. *arboreum* genome sequences respectively. Similarly, the assembled transcripts were BLASTN against the *G*. *raimondii* and *G*. *arboreum* genome sequences with only the top BLASTN result kept to determine their location. The location of differentially expressed transcripts were then compared with the location of SSR markers, to identify the differentially expressed R-genes resided 1megabase within the SSR markers mapped loci.

### Verification of RNA sequencing results by quantitative real-time PCR

For qRT-PCR, new RNA samples were collected following the same experimental procedures used in RNA-seq (i.e. same plant materials, stress treatment, and root sample collection time). cDNA was prepared from newly collected total RNA using Quanta qScript cDNA supermix and the cDNA was diluted before it was used for analysis by qRT-PCR. QRT-PCR was peformed with SYBR-Green Supermix in an Eppendorf Mastercycler ep realplex. Each reaction contained 4μl cDNA template, 9μl SYBR-Green supermix, 1μl of 10μm forward and reverse primers, and 4μl sterile water. The qRT-PCR program consisted of one cycle at 95°C for 20 sec, followed by 40 cycles of 15 sec at 95°C, 20 sec at 60°C, and 30 sec at 68°C. The last step for each reaction was melting curve generation to test the amplicon specificity. All qRT-PCR reactions were performed in three technical and two biological replicates. All samples were compared with the internal reference gene *PP2A* (catalytic subunit of protein phosphatase 2A) [96]. The primer sequences for the genes that were verified through qRT-PCR are listed in (S5 Table). Student t-test was used to calculate the statistical significance (P-values).

## Supporting Information

S1 Fig Experiment design flow chart. RN: reniform nematode; U: reniform nematode uninfested; I: reniform nematode infested. * Total RNA generated from combined reniform nematode infested cotton root samples at 1DPI (days post infestation), 3DPI, 5DPI, and 10DPI. Root pictures are representatives of reniform nematode infested and uninfested roots from corresponding genotypes. Blue and red arrows correspond to reniform nematode infested and uninfested samples respectively.
(TIF)

S2 Fig Number of differentially expressed transcripts between genotypes without *R*. *reniformis* infestation. (TIF)

S3 Fig Chromosome distribution of *R*. *reniformis*-responsive *R*-gene annotated transcripts. (A) Chromosome distribution of *R*. *reniformis*-responsive *R-*gene annotated transcripts on A2 (*G*. *arboreum*) and *D5* (*G*. *raimondii)* subgenomes; (B) Chromosome location of *R*. *reniformis*-responsive *R-*gene annotated transcripts on Chromosome 4 of A2 (*G*. *arboreum*) subgenome; (**C)** Chromosome location of *R*. *reniformis*-responsive *R-*gene annotated transcripts on Chromosome 7 of D5 (*G*. *raimondii*) subgenome. * Each dot represents a single *R*. *reniformis*-responsive *R-*gene annotated transcript on figure B and C.
(TIFF)

S4 Fig qRT-PCR analysis of 9 selected genes. The y-axis indicates relative expression compared with internal reference gene *PP2A*, the x-axis indicates genotype and treatment type. The numbers above each bar indicate RPKM (reads per kilobase of transcript per million mapped reads) values of each transcript in the corresponding genotype/treatment. Red color highlights significantly differentially expressed transcripts pairs (Fold change value *>* 2 & FDR P-value < 0.01) as determined by statistical analysis of RNA-seq data. Asterisks indicate transcripts that were significantly (P-value < 0.05) differentially expressed identified by qRT-PCR. qRT-PCR data was calculated using the **Δ**Ct method, and student t-test was used to determine P-value. The relative expression data represent means of two biological replicates, and error bars represent standard error of biological replicates.
(TIFF)

S5 Fig Transcriptome data analysis pipeline. DS: DP90 and SG747; L1: LONREN-1; B713: BARBREN-713; U: reniform nematode uninfested; I: reniform nematode infested; DEG: differentially expressed genes/transcripts
(TIFF)

S1 Table The number of genes/transcripts expressed in each library. (XLSX)

S2 Table List of differentially expressed transcripts close to the *R*. *reniformis* resistance QTLs. (XLSX)

S3 Table List of differentially expressed *R-*gene annotated transcripts between uninfested libraries. (XLSX)

S4 Table List of *R*. *reniformis*-responsive *R*-gene annoated transcripts. (XLSX)

S5 Table Transcripts and primers used for qRT-PCR verification. (XLSX)
