# Supplementary figures and images for: Transcriptome Analysis of Cotton (Gossypium hirsutum L.) Genotypes That Are Susceptible, Resistant, and Hypersensitive to Reniform Nematode (Rotylenchulus reniformis)
Source: PLoS One. 2015 Nov 16;10(11):e0143261. doi: 10.1371/journal.pone.0143261 (PMC4646469; doi:10.1371/journal.pone.0143261)

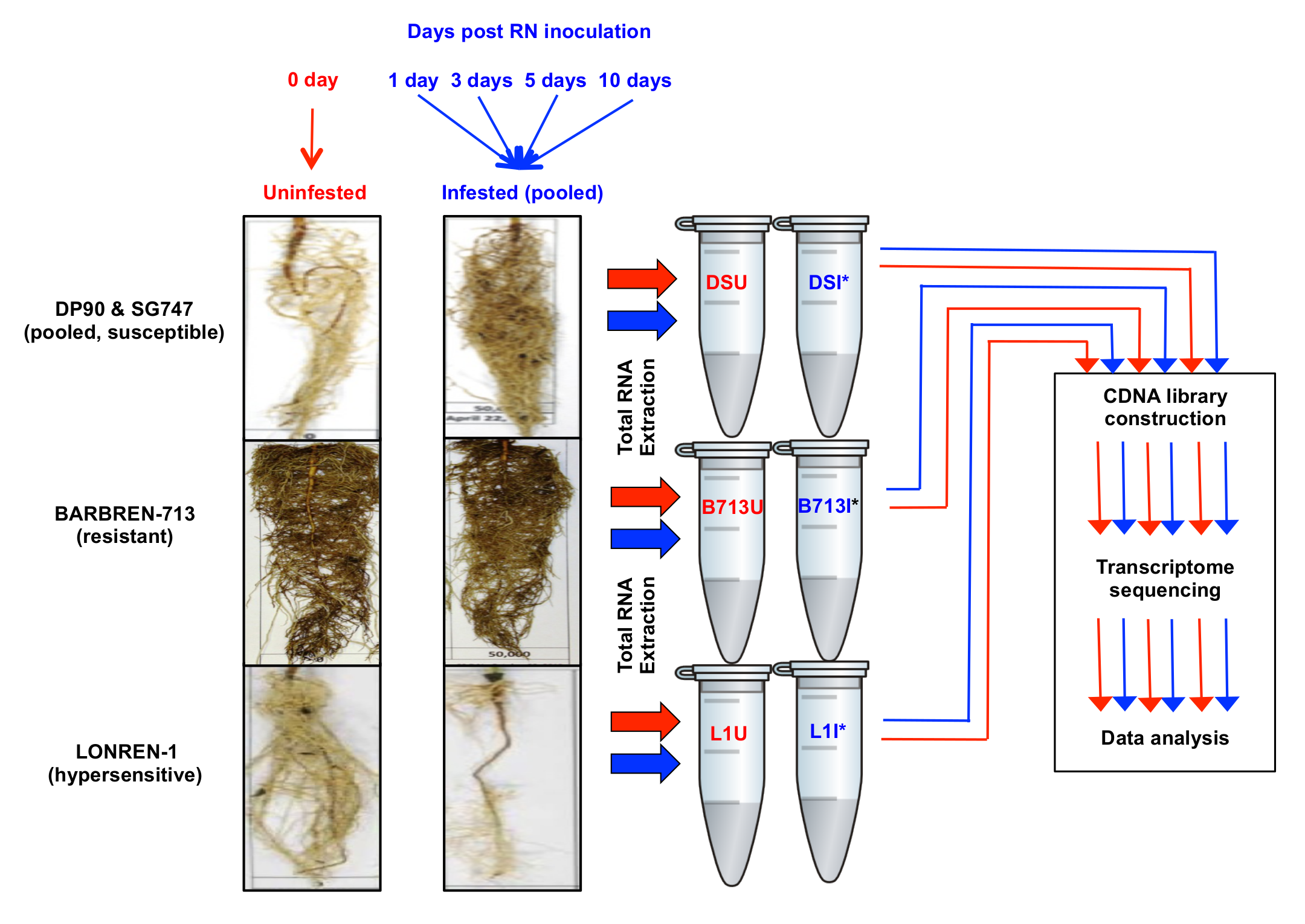

Supplement: S1 Fig — RN: reniform nematode; U: reniform nematode uninfested; I: reniform nematode infested. * Total RNA generated from combined reniform nematode infested cotton root samples at 1DPI (days post infestation), 3DPI, 5DPI, and 10DPI. Root pictures are representatives of reniform nematode infested and uninfested roots from corresponding genotypes. Blue and red arrows correspond to reniform nematode infested and uninfested samples respectively. (TIF) [file pone.0143261.s001.tif]

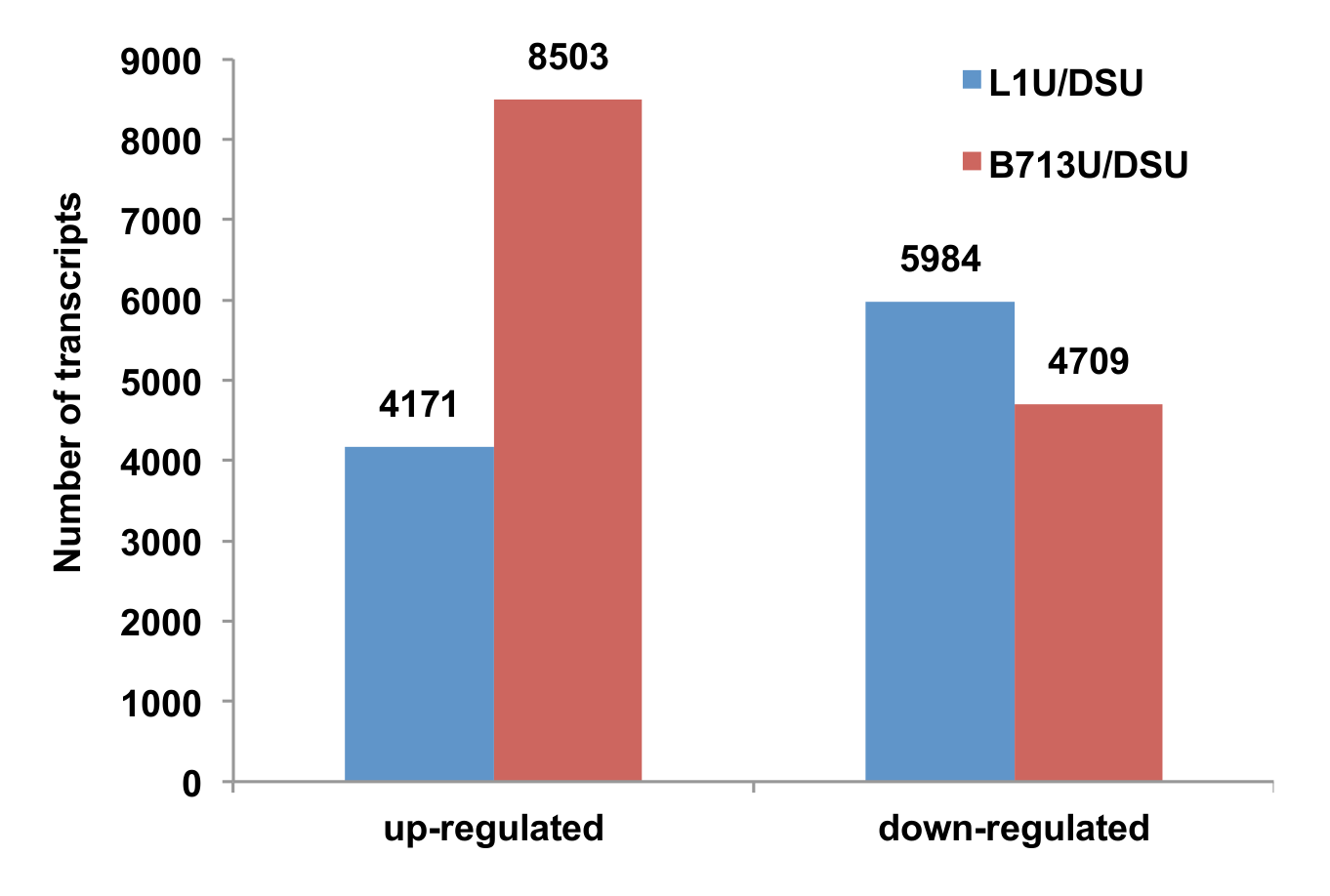

Supplement: S2 Fig — (TIF) [file pone.0143261.s002.tif]

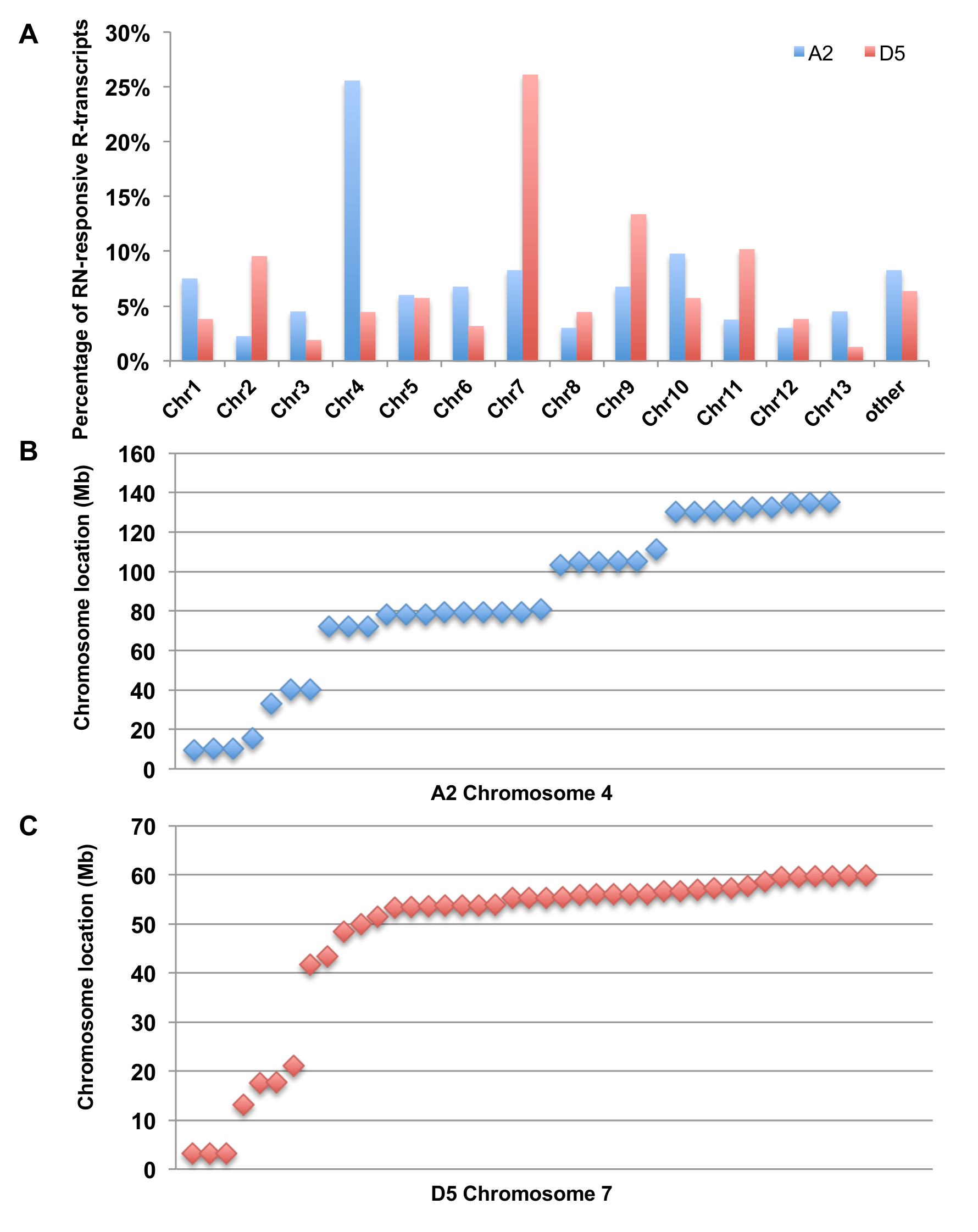

Supplement: S3 Fig — (A) Chromosome distribution of R. reniformis-responsive R-gene annotated transcripts on A2 (G. arboreum) and D5 (G. raimondii) subgenomes; (B) Chromosome location of R. reniformis-responsive R-gene annotated transcripts on Chromosome 4 of A2 (G. arboreum) subgenome; (C) Chromosome location of R. reniformis-responsive R-gene annotated transcripts on Chromosome 7 of D5 (G. raimondii) subgenome. * Each dot represents a single R. reniformis-responsive R-gene annotated transcript on figure B and C. (TIFF) [file pone.0143261.s003.tiff]

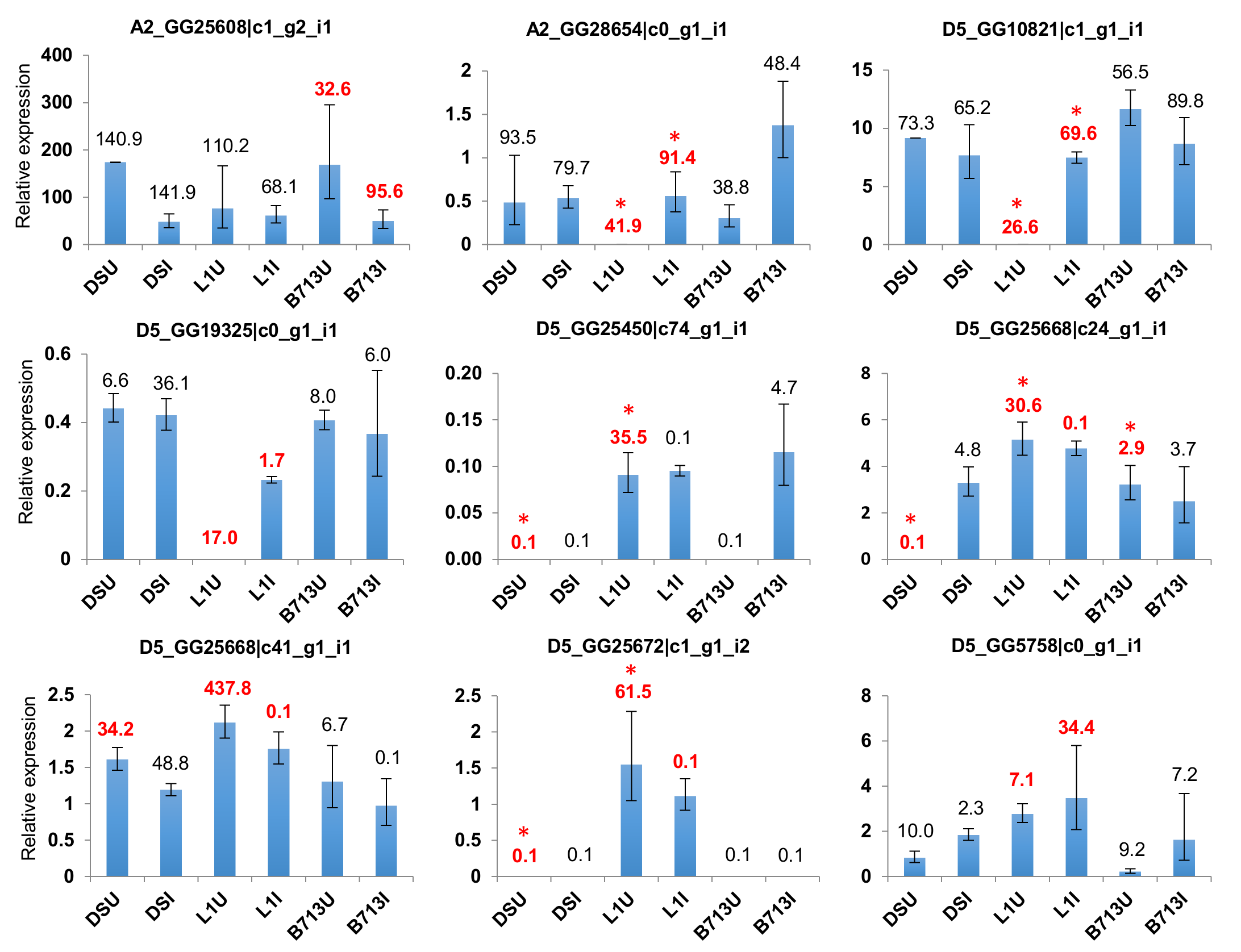

Supplement: S4 Fig — The y-axis indicates relative expression compared with internal reference gene PP2A, the x-axis indicates genotype and treatment type. The numbers above each bar indicate RPKM (reads per kilobase of transcript per million mapped reads) values of each transcript in the corresponding genotype/treatment. Red color highlights significantly differentially expressed transcripts pairs (Fold change value > 2 & FDR P-value < 0.01) as determined by statistical analysis of RNA-seq data. Asterisks indicate transcripts that were significantly (P-value < 0.05) differentially expressed identified by qRT-PCR. qRT-PCR data was calculated using the ΔCt method, and student t-test was used to determine P-value. The relative expression data represent means of two biological replicates, and error bars represent standard error of biological replicates. (TIFF) [file pone.0143261.s004.tiff]

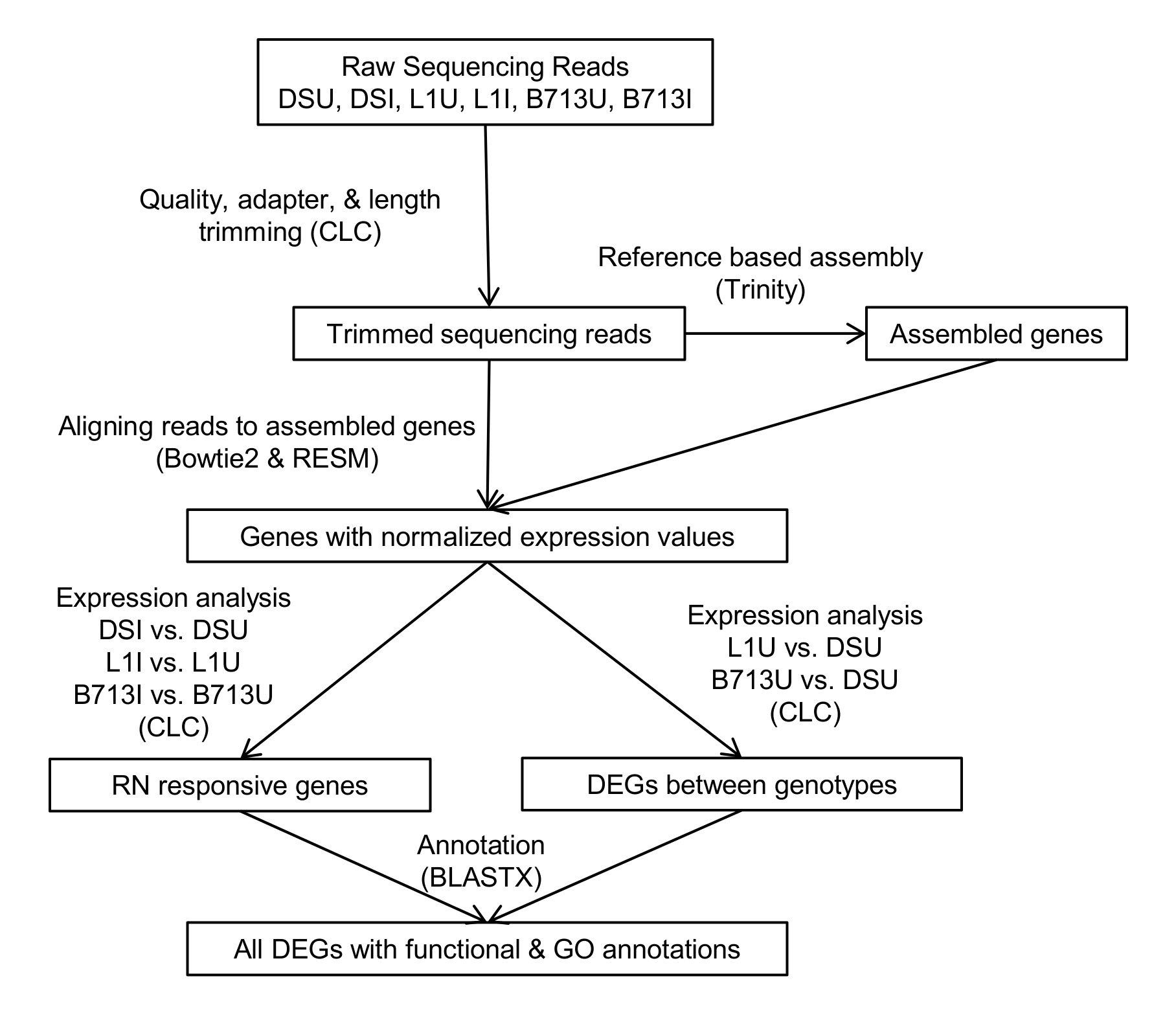

Supplement: S5 Fig — DS: DP90 and SG747; L1: LONREN-1; B713: BARBREN-713; U: reniform nematode uninfested; I: reniform nematode infested; DEG: differentially expressed genes/transcripts (TIFF) [file pone.0143261.s005.tiff]
